# Advancing the adoption of oncology decision support tools in Europe: insights from CAN.HEAL

**DOI:** 10.3389/fdgth.2026.1784519

**Published:** 2026-03-27

**Authors:** Nancy Frederickx, Guy Froyen, Maud Kamal, Célia Dupain, Matteo Pallocca, Julie Maetens, Nikolas von Bubnoff, Gennaro Ciliberto, Pauline De Wurstemberger, Zeina Chamoun Morel, Rossana Alessandrello, J. Matt McCrary, Brigitte Maes, Ruggero De Maria, Frédérique Nowak, Jose Maria Castellano-Garcia, Claudia Prats, Patrizio Giacomini, Aline Hebrant, Gordana Raicevic Toungouz, Marc Van den Bulcke, Els Van Valckenborgh

**Affiliations:** 1 Cancer Centre, Department of Epidemiology and Public Health, Sciensano, Brussels, Belgium; 2 Laboratory for Molecular Diagnostics, Jessa Hospital, Hasselt, Belgium; 3 Faculty of Medicine and Life Sciences, LCRC, University of Hasselt, Hasselt, Belgium; 4 Department of Drug Development & Innovation (D3i), Institut Curie (IC), Paris, France; 5 IHU PRISM National Precision Medicine Centre in Oncology, Gustave Roussy, Paris, France; 6 Institute of Endotypes in Oncology Metabolism and Immunology “G. Salvatore” (IEOMI-CNR), Naples, Italy; 7 Department of Hematology and Oncology, University Medical Center Schleswig-Holstein (UKSH) and University Cancer Center Schleswig-Holstein (UCCSH), Campus Lübeck, Lübeck, Germany; 8 IRCCS National Cancer Institute Regina Elena, Rome, Italy; 9 Advanced Training Office, Institut Curie (IC), Paris, France; 10 Innovation & Strategic Futures Area, Agency for Health Quality and Assessment of Catalonia (AQuAS), Barcelona, Spain; 11 Department of Human Genetics, Hannover Medical School, Hannover, Germany; 12 Institute of Clinical Genetics and Genomic Medicine, University Hospital of Würzburg & University of Würzburg, Würzburg, Germany; 13 Alleanza Contro il Cancro, Rome, Italy; 14 Dipartimento di Medicina e Chirurgia Traslazionale, Università Cattolica del Sacro Cuore, Rome, Italy; 15 Fondazione Policlinico Universitario Agostino Gemelli IRCCS, Rome, Italy; 16 Health Technologies Institute, Inserm, Paris, France; 17 UOSD Medicina di Precisione in Senologia, Fondazione Policlinico Universitario Agostino Gemelli IRCCS, Rome, Italy

**Keywords:** AI data-driven precision oncology, CAN.HEAL, clinical decision system, data integration, decision support tool (DST), digital framework, Molecular Tumour Board, personalised oncology

## Abstract

Effective cancer care increasingly depends on digital decision support tools (DSTs) to interpret complex clinical, molecular, and genomic data and guide personalised treatment decisions. However, the oncology DST (oncDST) landscape remains fragmented, with limited interoperability, inconsistent standards, and uneven clinical adoption across healthcare systems. This fragmentation hinders routine clinical use and impedes the demonstration of robust clinical benefit. To address these challenges, the CAN.HEAL consortium proposes the EU-oncDST digital framework, a conceptual, harmonised, interoperable, and modular architecture designed to integrate existing oncDSTs across Europe. Developed through consortium-wide consultations, an EU-level survey and comprehensive mapping of both public and private solutions, the framework provides a practical pathway for implementing interoperable oncDSTs while fostering stakeholder collaboration and innovation. It also promotes the improvement of data-driven precision oncology, highlighting the integration of artificial intelligence, enabling continuous patient follow-up, and supporting the development of a learning cancer system. At its core, the framework empowers Molecular Tumour Boards (MTBs) to operate efficiently at institutional, national, and European levels. By offering a harmonised, interoperable, and modular architecture designed to integrate clinical, molecular and genomic data, the framework strengthens evidence-based and personalised treatment recommendations. A phased action plan links MTB deployment to the implementation of oncDSTs. Early phases focus on piloting and validating oncDST use within MTBs, optimising patient-centred consultations, harmonising variant annotation, and enhancing clinical trial matching. Overall, the EU-oncDST digital framework aims to provide a practical and collaborative pathway to strengthen oncology decision-making and accelerate the translation of precision medicine into clinical benefit across Europe.

## 1 Introduction: the state-of-the-art in oncology decision support tools

Cancer is a complex and heterogeneous disease, with ongoing efforts to fully understand its underlying biology. It remains a major burden in Europe and is expected to become the leading cause of death by 2035 (1). Personalised cancer medicine, mainly driven by genomics, is predicted to reshape the management of the disease (2), acting across the entire patient continuum: prevention, screening, accurate diagnosis, access to state-of-the-art treatments, follow-up, and tertiary prevention. As the field continues to shift toward personalised care, the continuous emergence of new insights and therapies adds a significant cognitive burden to clinical decision-making. Clinicians must stay up to date with the growing number of publications and newly approved treatments (3), which have steadily increased over time (4).

To respond to this challenge, oncology Decision Support Tools (oncDSTs) have been developed as multifaceted electronic systems designed to integrate patient-specific data, clinical evidence, and other relevant health information to provide tailored recommendations and support clinicians in applying their expertise at the point of care, ultimately promoting more informed and individualised treatment choices (3, 5–7). Such solutions are being implemented across various stages of the patient care continuum (8–13).

The most promising application of oncDSTs is in guiding precision oncology through the integration of molecular data into clinical decision-making by synthesising complex genomic and molecular information with evolving clinical evidence (9, 10). One key area where oncDSTs prove valuable is in Molecular Tumour Boards (MTBs), multidisciplinary teams that review complex molecular profiles and provide personalised treatment recommendations based on tumour-agnostic criteria to identify targeted therapies and clinical trials tailored to each patient's unique tumour profile and clinical history. Although the clinical benefit of MTBs needs to be further proven, they are becoming central to precision oncology, and the demand for enhanced digital support in discussions and reporting is becoming increasingly critical. Real-world experience indicates that MTBs enhance molecular insights to refine diagnoses and improve clinical trial awareness. They also improve confidence in interpreting genomic data and provide significant educational benefits (14). In addition, national clinical studies reinforce the implementation of MTBs, combined with customised approaches to reviewing patient cases within MTB discussions (15, 16), using homemade interactive web dashboards or defined workflows through publicly available databases such as ClinVar (17) and OncoKB™ (18). Recently published MTB guidelines recommend the development of structured platforms for virtual MTB consultation with secure data management, integration of clinical and research data, trial matching, outcome tracking, support from artificial intelligence-machine learning algorithms, and compliance with European Electronic Health Record Exchange Format (EHRxF), all supported by robust cybersecurity (19, 20). Pallocca et al. (2024) reinforced oncDSTs' utility by outlining digital tools and expertise required to interpret genomic data and guide biomarker-driven therapies, providing the technical basis for virtual MTB operations at all levels (21).

Despite the growing promise of oncDSTs, real-world implementation has revealed significant challenges. One example is IBM Watson for Oncology (WFO), developed in collaboration with Memorial Sloan Kettering Cancer Centre. WFO was introduced in multiple countries to support cancer treatment decisions (22). Concordance studies have generally shown high agreement between WFO and human decision, particularly in breast, colorectal, gastric, and prostate cancers (22–24). However, practical use has faced challenges, such as a lack of retrospective learning, limited training data for some cancer types (e.g., urological) and missing patient-specific factors or ethnic variations. Additionally, WFO relied exclusively on U.S. Food and Drug Administration (FDA) approved drugs and National Comprehensive Cancer Network (NCCN) guidelines, which are specific to the U.S. market and did not account for other international practices or reimbursement policies, limiting its ability to be effectively implemented elsewhere (24, 25).

The limitations observed with WFO are not unique. Comparative studies of commercial and publicly available oncDSTs have highlighted critical issues that must be considered in clinical settings (26–28). While these solutions offer a time-saving and reproducible approach from variant annotation until reporting, comparison studies also revealed discrepancies in strategic and interpretive frameworks, resulting in varying somatic variant annotation and interpretation and, in the end, different treatment recommendations (26, 27). Altogether, the findings point to a persistent lack of standardisation in annotation methods, interpretation models, and matching algorithms, highlighting the pressing need for harmonisation to ensure consistency, reliability, and clinical applicability in oncology.

To confront the challenges, cancer centres and academic institutions across Europe joined forces to implement a conceptual framework to support harmonisation and interoperability of oncDSTs, enabling evidence-based decision-making across Europe. This effort was part of CAN.HEAL (https://canheal.eu/), an action grant project (Nov 2023-Apr 2025) under the EU4Health Programme with 47 cancer centres and academic institutions across 17 countries participating. Its main objective was to improve access to cancer prevention, diagnosis, and treatment for individuals, patients and survivors through personalised medicine. The project aimed to enhance innovation in cancer diagnosis and treatment by providing guidelines and recommendations. One of the focuses of the consortium was on advancing and strengthening the oncDST field and MTB operations across institutional, national, and European levels, paving the way for future implementation that addresses the patient care continuum and meets the real-world needs of clinicians in Europe. Within this framework, a dedicated work package (WP10) analysed current commercial and publicly available oncDST solutions for molecular profiling in cancer diagnostics and treatment, identifying gaps and opportunities. Described further in this paper, the EU-oncDST digital framework represents the main outcomes of this effort, providing a structured approach for harmonisation, interoperability and demonstrating the clinical benefit of oncDSTs (29, 30).

## 2 Materials and methods

**Figure 1:**
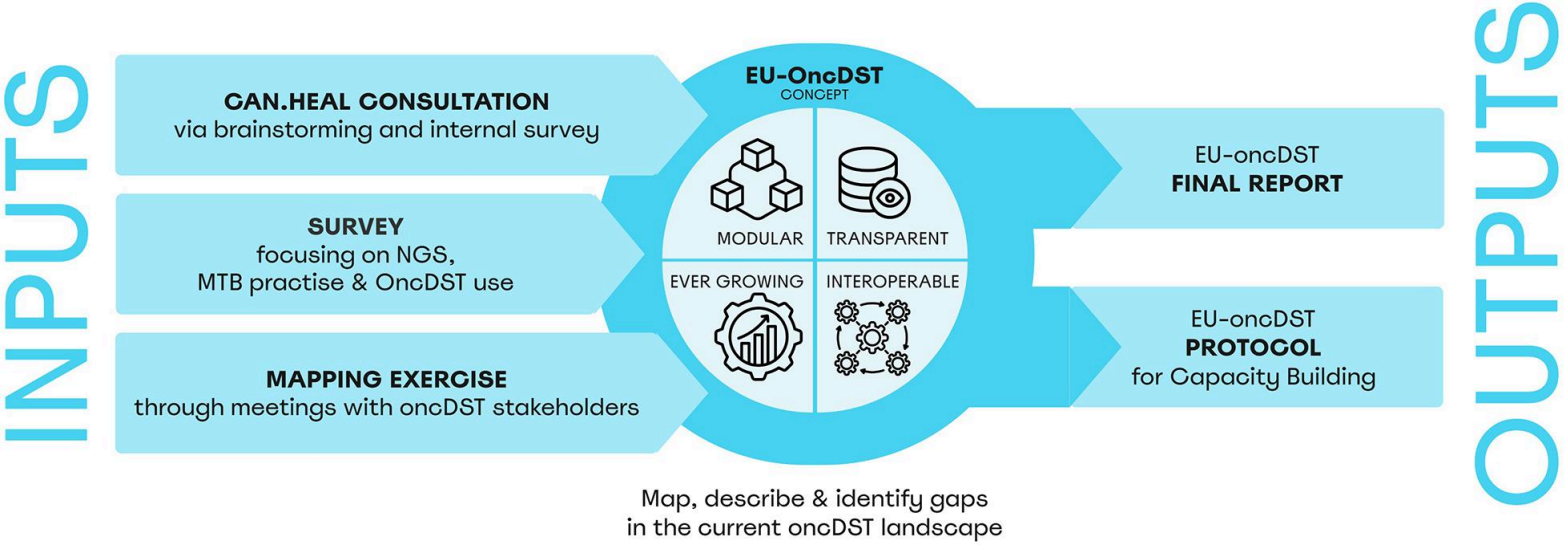
Overview of the EU-OncDST concept development and its methodology. The figure illustrates the relationship between inputs and outputs, highlighting the CAN.HEAL consultation, survey, and mapping exercise as primary inputs. These lead to a modular, transparent, interoperable and ever growing digital framework, resulting in a final report and protocol for capacity building. Icons from: “Module” by Anil, “Database view” by Vectorstall, “Improvement” by Ali Nur Rohman and “Interoperability” by Squly_icon, licensed under Royalty-Free License.

Diverse activities for exploring the oncDST landscape were conducted within the CAN.HEAL project. These included CAN.HEAL consortium-wide consultations, a comprehensive survey on next-generation sequencing (NGS), MTB and oncDST practices in the EU and a mapping exercise (Figure 1).

The CAN.HEAL consultations defined the criteria for eligible solutions, which were required to provide a DST designed to translate NGS and relevant clinical data to support MTB decision-making. These consultations also established the framework and objectives for each activity, including a process to objectively review tool features and capabilities. The emerging consortium criteria collected during the consultations led to a “tool assessment sheet” to guide the interviews planned for the mapping exercise, enabling comprehensive assessment and standardised recording. Furthermore, feedback was gathered during the consortium consultations at key steps of the EU-oncDST digital framework development.

A total of 19 oncDST solution providers were identified through the CAN.HEAL network and complementary desk research and invited to participate in the mapping exercise. Among these, 13 from both private and public institutions participated, while 7 did not respond, primarily due to the absence of identifiable contacts. No solution provider explicitly declined participation.

The mapping exercise involved interviews, presentations, and/or demonstrations; each exchange was recorded using the tool assessment sheet. These interviews occurred from June to November 2023. In parallel, the comprehensive survey collected 116 responses across the EU, with 75 reaching the oncDST section. Of these, 11 respondents reported using an oncDST. These respondents held roles such as clinicians, molecular biologists involved in diagnostics, and laboratory medicine specialists. Their insights were analysed and incorporated into the mapping exercise. Analysis of the mapping exercise and survey refined the emerging consortium criteria. A validation exercise via an online survey invited solution providers to indicate alignment with the criteria, categorising responses as “align,” “partially align,” or “not align,” with justifications. Nine of the 13 (69%) stakeholders completed this survey; individual responses remain confidential. Together, these activities enabled the development of the EU-oncDST digital framework. Full methodological details, including the list of tools assessed, the tool assessment sheet and survey questions, are available in the corresponding project deliverables (29, 30).

## 3 Results

### 3.1 Cross-cutting observations from the mapping exercise

Rather than conducting direct tool comparisons, the CAN.HEAL consortium concentrated the mapping exercise on identifying shared features, common observations, and potential elements of improvement across the solutions (Table 1). The mapping exercise revealed several cross-cutting observations regarding data entry, integration, analytical capabilities, and interoperability of existing tools.

**Table 1:** An overview of the description, observations and recommendations for the distinct modules within the EU-oncDST digital framework.

Modules	EU-oncDST Digital Framework Description	CAN.HEAL Observations	Dependencies	Recommendations
**EHR/ LIMS** 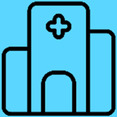	Provides patient data Connects to the existing solution in place Allows automatic interoperability Collects MTB reports	In place to support patient care, streamline processes, and enhance data accuracy 71% of survey respondents still rely only on the hospital tool to support MTB discussion (CAN.HEAL Survey) Large variety of local EHR/LIMS	Patient data module Final Report	Leverage existing solutions to support the EU-oncDST digital framework
**Patient case building** 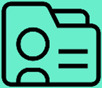	Fuses outputs from Patient Data, Bioinformatics, and Clinical Recommendation Modules into a unified and structured report for the MTB Module	Current oncDST solutions provide a report, but the information can vary from one solution to another Customisation is possible before the edition in PDF	Patient data Bioinformatics Clinical recommendations MTB	Report harmonisation with a consistent format regardless of the solution used Guideline alignment—meets patient data requirements for MTB discussions as per CAN.HEAL (19) and ESMO (20) Align with MTB guidelines to meet patient data requirements
**Patient Data** 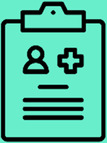	Integrates automatically: ◦ Patient data integration: demographics, medical history ◦ Test results integration: pathology, imaging data ◦ Research data integration: germline alterations, polygenic risk scores, pharmacogenomics Supports EHR interoperability Complies with: European EHR exchange format & European Health Data Space (EHDS)	Manual entry of patient data Minimal demographic information No systematic integration of test results Limited interoperability	EHR/LIMS Patient case building case	Enhance interoperability with EHR/LIMS Automate streamlined data exchange and processing Integrate a unified common data model and language system Incorporate test results
**Bioinformatics** 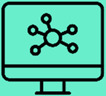	Involves comprehensive annotation & classification of genomic and transcriptomic data for actionable diagnosis, prognosis, and treatment insights Processes diverse biomarkers: SNVs, indels, CNVs, gene fusions, exon-skipping events, MSI, TMB, HRD, mutation signatures, variants of unknown significance (VUS), and multi-omics alterations Aligns with guidelines for evidence-based interpretation (e.g., ACMG-AMP, ESCAT) Is flexible to customise with guidelines selection in line with the national or regional situation Is transparent	Most advanced module with various solutions Lack of comprehensive analysis of multi-omics alterations Insufficient VUS assessment No integration with pharmacogenomics and germline data Lacks AI-driven tools for holistic precision oncology Discrepancies in tool annotations and classification	Patient building case Clinical recommendation	Resolve annotation discrepancies across tools to ensure harmonisation and consistency, enabling development of a unified, advanced, and ethically governed cancer learning system Promote an AI-driven and transparent tool to support a precision oncology approach Harmonise tool annotations and classification
**Clinical recommendations** 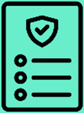	Integrates diagnosis & prognosis interpretation, treatment recommendation, and clinical trial availability Connects with drug-genomic interaction databases: ClinVar, ClinGen, OncoKB, CiVIC, JAX CKB Adapts to local clinical guidelines and reimbursement schemes Ensures transparency through evidence-based data connections Incorporates patient data, genomic profiles analysis and patient preferences for personalised recommendations using AI/ ML Matches automatically patient-specific clinical trials based on patient data and genomic profile Highlights trial eligibility, locations, contact info, and enrolment availability Prioritises treatment and trial options	Lack of tumour-agnostic analysis and prognostic insights Absence of automated clinical trial matching incorporating patient data. Most solutions integrate ClinicalTrials.gov Integration of national drug repositories is achievable through tool provider collaboration References embedded in databases, not always directly accessible Extensive reference or trial lists requiring user proficiency for effective filtering Treatment prioritisation based on evidence levels Trial preselection by phase (≥ Phase 2) or recruitment status (open) is available in some solutions	Patient building case Patient data Bioinformatics	Prioritise automatic integration of patient-specific clinical and genomic data for personalised treatment plans Improve and develop tumour-agnostic approaches and prognostic insights Enhance diagnostic interpretation, including for cancers of unknown primary (CUP) Promote integration of national drug repositories linked to reimbursement schemes Develop advanced AI/ML systems that integrate patient data, diagnostic results (MRI, CT, pathology), genomic actionability, and patient preferences to prioritise treatment recommendations Implement automated clinical trial matching based on genomic and clinical data
**MTB** 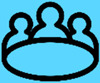	Allows centralised decision-making through data-driven discussions for accurate, efficient, and personalised oncology care Integrates comprehensive data by consolidating clinical data, genomic profiles, treatment options, and clinical trial availability into a single platform Structures reporting to produce standardised MTB reports for physicians, integrates them into EHR systems, and supports the Data-Driven Precision Oncology module Includes a query functionality in Data-Driven Precision Oncology modules to analyse previous cases Includes links to guidelines and literature MTB–oncDST synergy by incorporating MTB feedback to refine oncDST functionalities, ensuring alignment with evolving clinical practices. Adheres to MTB Guidelines (19, 20)	Limited operational solutions Accelerates MTB discussions and reporting when available No query feature for follow-up data, limiting retrospective analysis Lack of a structured MTB report	Patient Building Case Final Report Patient Consultation Data-Driven Precision Oncology	Develop in collaboration with MTB members Support the development of the Data Driven precision oncology module Tailor to operational level, institutional, national, European Support patient counselling
**Final report** 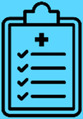	Summarises MTB recommendations in a structured format Includes digital Implementation enabling integration into EHR/LIMS and Data-Driven Precision Oncology modules after pseudonymisation Supports patient counselling by including relevant information to guide discussions with patients	Lack of report harmonisation Limited interoperability	MTB EHR/LIMs Data-Driven Precision Oncology Patient Consultation	Develop patient counselling support for the Patient Consultation module Enhance interoperability to ensure seamless data exchange across modules Harmonise structured formats for consistent reporting in alignment with MTB guidelines
**Patient consultation** 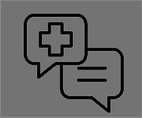	Enhances patient engagement and education on genetic mutations, treatment options, and clinical decisions Empowers patients to participate in their care actively and set expectations Facilitates informed consent for data sharing and clinical trial enrolment Supports the Follow-up module with patient-reported outcome measures/patient-reported experience measures (PROMS/PREMS)	Was not considered in the mapping exercise	MTB Final report Follow-up	Complementary investigation to establish the strategy
**Data-Driven Precision Oncology** 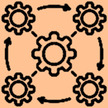	Integrates the Learning Cancer System, Follow-up, and Digital Twins modules for a comprehensive, data-driven approach to personalised oncology care Uses AI/ML to analyse pseudonymised structured MTB reports and real-world evidence (RWE) for patient tracking, outcome prediction, and treatment guidance Includes feedback loop across the Learning Cancer System, Follow-up, and MTB modules using patient data to improve cancer understanding and treatment decisions Digital Twins provide dynamic, real-time representations of patient health for predictive insights and active treatment management. Adheres to findable accessible interoperable reusable (FAIR) principles, EHDS recommendations, and EU regulations (GDPR, IVDR, MDR, AI Act) to ensure data security and compliance	Limited interoperability infrastructure Query feature not available	MTB Final report Follow-up Learning cancer system Digital twin Patient consultation	Use AI as a collaborative partner to enhance evidence synthesis, patient stratification, and precision decision-making Ensure AI interpretability and transparency Address privacy and data-sharing challenges Achieve interoperability with hospital systems Secure regulatory approval for clinical use Implement follow-up modules to monitor performance and update recommendations Build on previous ongoing initiatives for data harmonisation and access

Icons from: “Hospital” by Colourcreatype, “Patient file folder” by Naufal Hudallah, “Patient Data” by henry, “Biological informatic” by Soremba, “Guidelines” by Zky Icon, “Meeting” by romzicon, “Medical report” by NAPISAH, “Medical Consultation” by Made x Made and “Interoperability” by Squly_icon, licensed under Royalty-Free License.

Patient information is mostly entered manually, with automatic integration limited by heterogeneous EHR/LIMS systems, although integration is easier within clinical trial settings due to data standardisation. Demographic information can be added and customised according to institutional preference. Some tools can integrate prior test results, such as laboratory data, tumour markers, organ function assessments, and biopsies, though medical imaging integration remains rare. At this stage, none of the reviewed tools enable the incorporation of patient demographic data or prior test results into analytical models supporting clinical recommendations.

The current market offers well-developed options for variant interpretation following secondary bioinformatic analysis encompassing annotation and classification of genomic alterations relevant to treatment recommendations. Most tools process SNVs, indels, CNVs, fusions, TMB, MSI, and HRD and flag variants of unknown significance (VUS), but do not yet support methylomic, transcriptomic, or proteomic alterations. The tools reference both international and national databases, combining AI support with manual curation, and classify variants according to ACMG, AMP, ESCAT, or custom tiers.

The tools automatically align variant annotation analyses with curated genomic datasets such as ESCAT (31), OncoKB^TM^ (18), or JAX-CKB^TM^ (32), which are commonly employed and offer options for regional customisation to align with regulatory frameworks (e.g., EMA, NICE, Health Canada, ESMO, NCCN, Swissmedic) though national drug regulations are not automatically integrated and may require technical collaboration with the provider. Analyses are generally tumour type specific and cannot yet be performed in an agnostic manner; the tools support diagnosis and treatment prioritisation based on levels of evidence, but do not automatically integrate patient-specific data for personalised therapeutic plans, do not reframe diagnostic hypotheses, or do not provide prognostic insights. Clinical trial matching is commonly available via ClinicalTrials.org integration, allowing filtered searches (e.g., by biomarker, geography, inclusion criteria, or trial phase), although the resulting lists are often extensive and require user expertise to navigate effectively.

All tools generate reports summarising patient data, variant interpretation, treatment options, and clinical trial matches, often accompanied by reference lists that are embedded but not always directly linked to specific recommendations; users have to manually review the list. Reports are typically available as customizable PDFs and are used to inform MTB discussions.

At the time of investigation, operational MTB dashboards were limited, with the most advanced solutions linked to clinical trial settings, such as the BALLETT app (Jessa hospital_V1) (15), Miracum consortium with the cBioPortal adapted_v6 APP (33–36), CGI-Clinics (37) and MTB-Portal (MTBP) (9), providing interactive overviews of patient cases discussed in MTB and supporting MTB reporting. Among the solutions reviewed, patient follow-up and query functions remain limited, although analytical data generated within tool hubs or institutional networks could provide insight from previous cases. The dashboards also allow the generation and editing of the MTB report, including MTB's recommendations, which subsequently inform the patient's clinician. Finally, regarding syntactic and semantic interoperability, infrastructure remains limited, with a few exceptions observed in tools linked to clinical trial or consortium implementations (15).

Complementing these observations, the comprehensive survey on NGS, MTB, and oncDST practices in the EU, together with internal CAN.HEAL practice, consistently reveals a significant gap in the adoption of oncDST across Europe. Most institutions continue to rely on hospital tools such as Electronic Health Record (EHRs) to support MTB discussions. A critical barrier to the implementation of oncDST is the lack of reimbursement, which limits its widespread use. Notably, participants often reported using a combination of tools (knowledge-based and non-knowledge-based), highlighting the absence of a comprehensive solution that adequately addresses all their needs. The survey specifically highlighted that the current strength of oncDST lies in NGS data analysis and interpretation, while its main weaknesses are the manual entry of information, labour-intensive processes, and the lack of automated treatment recommendations and prioritisation. This lack of automation could hinder the development of interoperability. A key takeaway from the survey is that all participants unanimously agreed that the recommendations provided by DSTs are beneficial in MTB discussions (29).

### 3.2 The EU-oncDST digital framework for consistent decision-making across Europe

To support harmonisation within the field, the CAN.HEAL consortium designed the EU-oncDST digital framework, comprising a concept and guidance. It addresses key challenges and limitations while facilitating oncDSTs deployment and implementation, as well as the demonstration of the tool's benefits. The framework provides a roadmap for implementing and enabling interoperability of existing oncDSTs, fostering stakeholder collaboration, and promoting innovation.

As agreed upon, it is envisioned as a comprehensive, modular, transparent, interoperable, and ever-growing system designed to support and enhance MTB activities (Figure 1). Its main intentions are to (i) centralise patient data, (ii) identify and evaluate actionable molecular alterations, (iii) offer personalised treatment recommendations, iv) facilitate clinical trial enrolment, (v) integrate genomic, clinical, and evidence-based data for MTB discussions, (vi) support patient counselling, and (vii) promote interoperability across centres to improve Data-Driven Precision Oncology (DDPO) within and across institutions enabling continuous patient follow-up and supporting the development of a learning cancer system.

**Figure 2:**
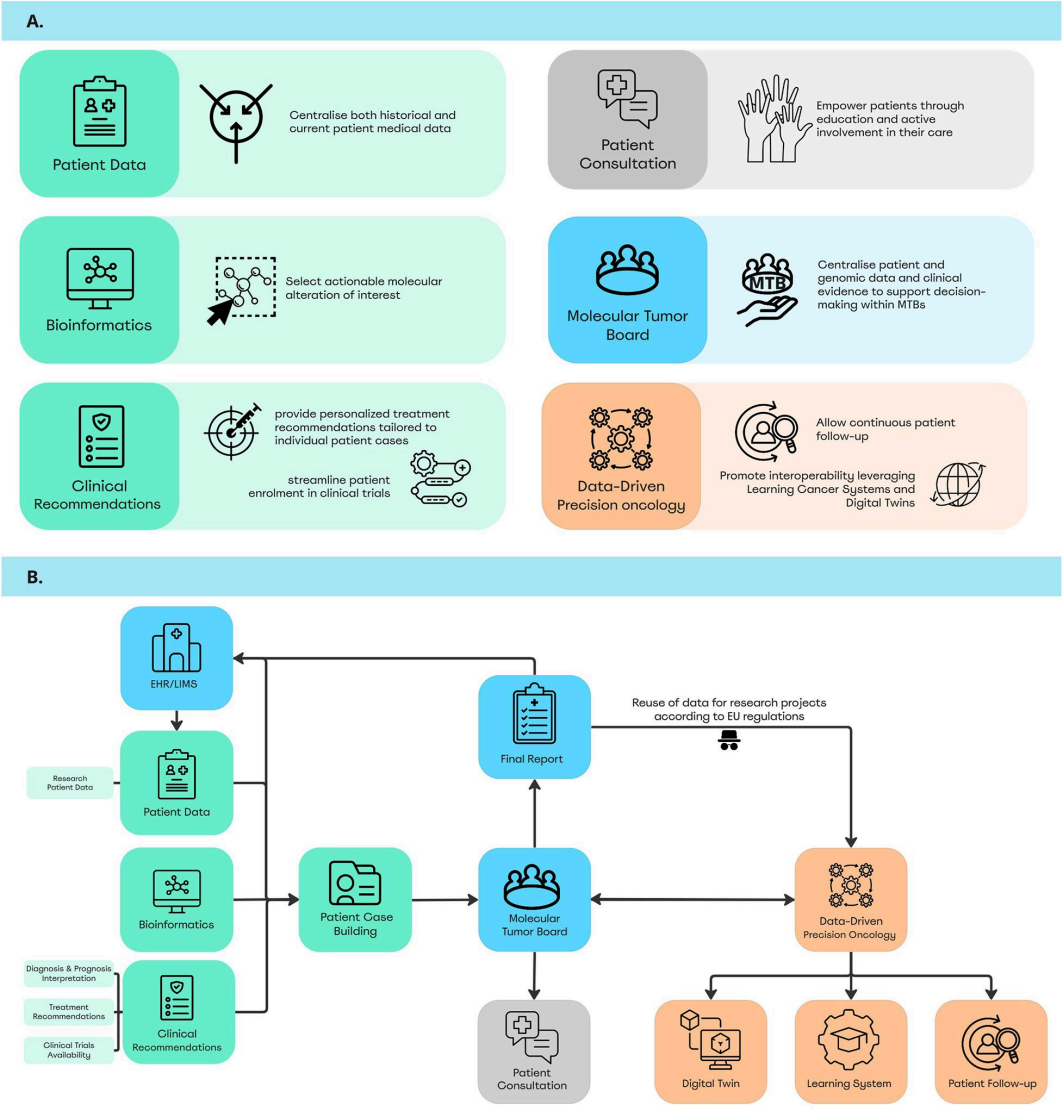
The EU-oncDST digital framework and its modules. **(A)** Purpose of the key distinct modules. **(B)** A detailed representation illustrating the interaction and interconnectivity between the modules. Icons from: “Patient Data” by henry,“Traffic” by Aidan Stonehouse, “Biological informatic” by Soremba, “Select” by Jaya99, “Molecular” by Fahrul Oktaviana, “Guidelines” by Zky Icon, “Precision medicine” by gravisio, “Streamline” by BEJOUN, Hand raised by ABDUL LATIF, “Hand” by revi abraham, “Meeting” by romzicon, “Follow up” by claretta, “Global” by nakals, “Learning system” by Anamika singh, “Digital twin” by Pentagon88, “Medical report” by NAPISAH, “Hospital” by Colourcreatype, “Anonymous” by Yuniarti Pahlevie, “Medical Consultation” by Made x Made and “Patient file folder” by Naufal Hudallah, licensed under Royalty-Free License.

The EU-oncDST digital framework is designed as a comprehensive system composed of multiple modules, each serving distinct yet interconnected purposes to support decision-making within MTBs (Figure 2A). The module interactions are illustrated in Figure 2B. When a patient is referred to the MTB, a digital case file is generated via the Patient Case-Building Module, which fuses data from the Patient Data Module connected to the patient's EHR/LIMS Module, Bioinformatics Module, and Clinical Recommendation Module. Once the case is ready for review, the MTB Module provides access to all relevant information for analysis. The MTB's recommendations are then compiled into a standardised Final report, which is sent to the patient's treating physician and stored in the EHR/LIMS. The MTB module also connects with the Patient Consultation Module to ensure patient understanding, involvement, and to obtain consent throughout the process. Upon receiving consent, the report is pseudo-anonymised in accordance with EU regulations for the reuse of data in research projects. The data are then integrated into the DDPO Module, which includes a Continuous Learning System, Patient Follow-up, and support for the development of the Digital Twin module. Data collected and processed within the DDPO module remain accessible via the MTB module for consultation, support, and guidance for the final recommendations. In addition, the follow-up module also feeds the patient consultation module to support, for instance, cancer registries. Table 1 provides an in-depth description of each module, its interaction with the overall framework, key observations and recommendations from the CAN.HEAL initiative for further development.

### 3.3 Recommendations and deployment

**Figure 3:**
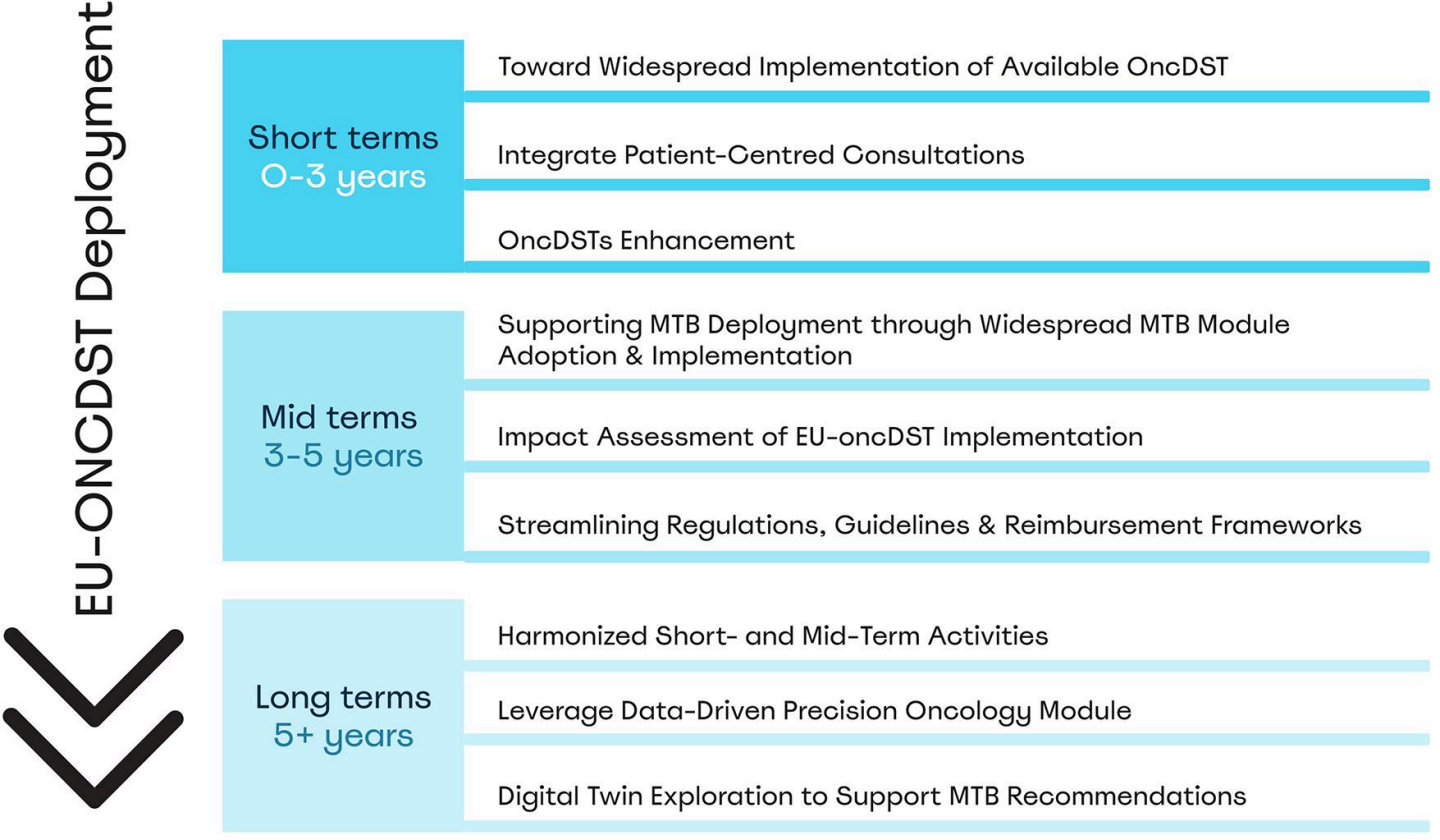
CAN.HEAL recommended phased deployment of the EU-oncDST digital framework for real-world implementation.

Building on the EU-oncDST digital framework, a deployment plan was proposed, including initial actions, areas for further investigation, and recommendations for use case applications and deployment opportunities (30). The key priority is to implement the EU-oncDST digital framework symbiotically with MTB, as these components are mutually reinforcing. OncDSTs enhance MTB discussions' efficiency, accuracy, and consistency by streamlining data integration, facilitating case review, and translating complex patient profiles into actionable insights, while MTB feedback helps refine oncDST functionalities. CAN.HEAL advocates a strategy that pairs oncDST implementation with MTB deployment, addressing operational barriers such as data entry burdens and tool reliability, while embedding these tools directly into MTB workflows. To support this, CAN.HEAL recommends a phased deployment strategy (Figure 3), ensuring that each step contributes to a cohesive, patient-centred, and future-proof learning system.

Short-term activities will focus on the broad implementation of the current oncDST in parallel with the deployment of MTBs. Real-world pilots aligned with MTB guidelines will demonstrate the added value of oncDSTs in improving discussion quality and patient outcomes, with MTB feedback driving ongoing refinement. An example is the Joint Action on Personalised Cancer Medicine (JA PCM; Nov 2025–Nov 2029) (38), which aims to strengthen the personalised cancer medicine network across Europe. Within this initiative, several pilots will be launched. One pilot will establish a transnational MTB network to improve access to standard-of-care and innovative treatment options for patients with rare or complex cases, thereby reducing disparities. Key goals include developing a legal and operational framework for cross-border MTB collaboration while ensuring alignment with existing MTB guidelines and promoting the use of oncDSTs.

Short-term enhancements also include variant annotation harmonisation and expansion to multi-omic, VUS, and cancers of unknown primary (CUP), improving clinical trial matching, enhancing interoperability and automation with EHR/LIMS systems, and advancing the interpretation of diagnosis and prognosis. Further details on specific enhancements are developed in the related deliverables (30). Additionally, further investigation on the format of patient-centred consultations, such as the EduCGI module under CGI-Clinics (37), aligned with established genetic counselling practices, should be performed. This will support informed consent and patient empowerment.

Mid-term activities focus on the widespread adoption and optimisation of the MTB module and its integration into clinical workflows. The module, which is still in its early stages, aims to reduce care disparities and improve access to expert guidance across Europe. Key enhancements include patient case visualisations, integration of diverse testing results, agnostic data handling, automated clinical trial matching, and structured MTB reports tailored to national contexts, all aligned with the EU-oncDST framework and MTB guidelines. This effort will be advanced through another JA PCM pilot dedicated to establishing continuous data collection via a data-sharing platform to reinforce MTB discussions. The platform aims to generate systematic real-world evidence for personalised cancer medicine across Europe by implementing a federated data-sharing model. This will enable harmonised aggregation of clinical and molecular data to support a European learning cancer system.

It is expected that AI will support this development by integrating diverse patient data, evolving evidence, and expert knowledge to support complex decision-making. Combining large language models (LLMs) with precision oncology tools and retrieval-augmented generation (RAG) improves decision accuracy and reliability compared to LLMs alone. Incorporating multimodal biomedical AI will further foster a learning cancer system and digital twins, accelerating personalised, real-time clinical guidance (39–41).

It is also important to keep in mind that these implementations will require investment in infrastructure, specialised personnel, cybersecurity, database maintenance, and quality assurance, but are expected to be cost-effective under the assumption that standardised, data-driven decision support will optimise care, reduce ineffective treatments, improve clinical productivity, and lower long-term healthcare costs. Regulatory and reimbursement frameworks must comply with GDPR, IVDR, MDR, and the AI Act, with early deployment data guiding standardised coverage. A regulatory sandbox, a controlled environment where new technologies can be tested and validated under regulatory supervision (42), can support safe testing of innovative AI solutions and provide a foundation for broader regulatory approval.

Finally, long-term goals aim to harmonise the short and mid-term activities leveraging the DDPO module. Ultimately, these efforts aim to optimise treatment, reduce costs, and ensure equitable access, transforming precision oncology into a sustainable healthcare model. Although some of these recommendations are being addressed through ongoing and upcoming EU initiatives such as JA PCM (38), EUnetCCC (43) and JA eCAN Plus (44), others will need to be taken forward through public–private partnerships (30) such as a pre-commercial procurement or through the innovative health initiative program that support the development, commercialisation and scaling of innovative solutions. Finally, across all of these initiatives, the inclusion of payers and patient groups will be essential to guiding implementation. Early engagement of payers can inform evidence requirements, reimbursement models, and workflow integration, while involvement of patients can strengthen understanding of needs, priorities, and meaningful outcomes.

## 4 Discussion

Our investigation on oncDSTs included 13 solutions, which were examined to illustrate the current strengths and limitations of the field. Multiple oncDSTs focusing on molecular profiling are already available and in operational use. These tools feature advanced bioinformatics modules with strong variant interpretation capabilities that utilise a wide array of established databases. Patient case reports are always used to support MTB discussions. Additionally, the use of oncDST fosters the development of a learning cancer system and promotes peer education, which are both important actions of the MTB. Reinforcing these strengths, all centres that reported using an oncDST in the survey recognised its clinical utility, noting that oncDST reports are consistently taken into account during MTB discussions and help optimise treatment selection and clinical trial enrolment by combining genomic, patient, and clinical information. These observations suggest that oncDSTs could improve patient outcomes by guiding more precise, data-driven treatment decisions, facilitating access to clinical trials, and supporting a learning cancer system that continuously collects and analyses comprehensive clinical and genomic data to refine diagnosis and treatment, which may, over time, positively influence survival and quality of life, though prospective studies are needed to confirm these effects and quantify their direct impact on survival and quality of life.

Despite these clear strengths and clinical value, several structural and operational challenges limit the broader implementation and effectiveness of oncDSTs across the EU. Although oncDST solutions feature options for customisation, such as integrating national guidelines and databases or connecting to local EHR/LIMS, their current implementation remains limited due to several structural and operational weaknesses across interconnected areas. Data integration and workflow automation remain major obstacles, as many tools still rely on manual, labour-intensive data entry and lack seamless integration with proprietary and publicly available systems. Data workflows are further constrained by insufficient automation, limited interoperability, and inadequate inclusion of critical data types such as methylomic, transcriptomic and proteomic profiles, as well as underrepresentation of diverse patient groups, including paediatric, geriatric and haematological cases. In addition, inconsistent variant classification and variability in annotation, interpretation, and treatment-matching algorithms, likely due to differences in software, evidence sources, and subjective guideline language, undermine the reliability and reproducibility of clinical recommendations, potentially widening disparities in patient care. Other limitations include insufficient support for MTB discussions, the absence of standardised reporting and diagnostic approaches, limited integration of AI-driven solutions, and inadequate consideration of national contexts in outputs. Regulatory complexities, particularly under GDPR and emerging AI laws, and reimbursement challenges further limit access and sustainable implementation across healthcare systems. Additionally, successful EU-wide oncDST deployment will require substantial financial investment, robust infrastructure, and specialised personnel, alongside rigorous quality control, cybersecurity, and transparency measures. Cost-effectiveness and trust in oncDST are essential for their widespread adoption and optimal use in clinical practice.

To leverage current weaknesses and support successful deployment, strategic solutions are needed, including federated data-sharing platforms, harmonised standards for reporting and interpretation, and AI-driven tools to enhance workflow efficiency. Future development should prioritise interoperability, validation of AI-assisted decision-making, and real-world evaluation to ensure reliability, build confidence among clinicians and patients, and progressively integrate oncDSTs into a sustainable, learning cancer system across Europe. Building on these deployment strategies, the EU-oncDST digital framework aims to pave the way for a truly integrated, patient-centred, and learning cancer system across Europe. Its successful EU-wide implementation will also depend on active collaboration across multiple stakeholders, including national healthcare authorities, regulatory bodies, industry partners, clinicians, and patient communities. Aligning oncDST development with existing EU initiatives, such as JA PCM and EUnetCCC, can facilitate standardisation, interoperability, and cross-border data sharing, while public–private partnerships can accelerate innovation, validation, and scaling of new tools. Patient engagement is critical to ensure that digital solutions meet real-world needs and support shared decision-making, while involvement of industry and payers is essential to establish sustainable reimbursement models. Together, this multi-stakeholder collaboration can transform the EU-oncDST digital framework into a truly integrated, patient-centred, and learning cancer system, capable of delivering equitable access to precision oncology across member states.

The findings described above reflect observations at the time the investigation was conducted. Since then, some solution providers have implemented significant updates. CAN.HEAL recognises the rapid pace of digital health advancements, so the results in this paper should be considered alongside more recent developments, in line with CAN.HEAL's vision of an ever-growing digital framework. In this dynamic context, continuous evaluation, iterative refinement, and adaptive implementation strategies combined with active stakeholder collaboration will be essential to ensure that oncDSTs remain clinically relevant, scalable, and capable of supporting a sustainable, learning cancer system across Europe.

To conclude, through consortium-wide consultations, an EU-level survey on NGS, MTBs, and oncDST practices, and a comprehensive mapping exercise, CAN.HEAL has designed the EU-oncDST digital framework to address the fragmented landscape of oncology decision-support tools in Europe. The framework offers a harmonised, interoperable, and modular architecture designed to integrate clinical, molecular, and genomic data, thereby strengthening evidence-based and personalised decision-making within MTB and facilitating the demonstration of the clinical benefits of oncDSTs. By supporting structured data collection, promoting interoperability across heterogeneous healthcare systems, and enabling equitable access to precision oncology, the EU-oncDST framework provides a practical foundation for a sustainable, learning cancer system. Its architecture anticipates future integration of AI-driven solutions, combining multimodal data and expert knowledge to further enhance clinical recommendations, clinician education, and peer learning. The findings of this study confirm that oncDSTs already demonstrate clear clinical utility when implemented, improving treatment selection and clinical trial matching. Fully realising their potential across Europe will, however, require coordinated deployment of the EU-oncDST framework, including strategic phased implementation, alignment with MTB development, and solutions for operational, regulatory, and reimbursement challenges. With sustained multi-stakeholder collaboration including clinicians, patients, industry, policymakers, and leveraging EU initiatives, the EU-oncDST digital framework can evolve into a truly integrated, patient-centred, and equitable precision oncology ecosystem, helping to advance high-quality cancer care for all patients.

## Data Availability

The original contributions presented in the study are included in the article/Supplementary Material, further inquiries can be directed to the corresponding author/s.
